# Performance of Language-Coordinated Collective Systems: A Study of Wine Recognition and Description

**DOI:** 10.3389/fpsyg.2016.01321

**Published:** 2016-09-27

**Authors:** Julian Zubek, Michał Denkiewicz, Agnieszka Dębska, Alicja Radkowska, Joanna Komorowska-Mach, Piotr Litwin, Magdalena Stępień, Adrianna Kucińska, Ewa Sitarska, Krystyna Komorowska, Riccardo Fusaroli, Kristian Tylén, Joanna Rączaszek-Leonardi

**Affiliations:** ^1^Institute of Computer Science, Polish Academy of SciencesWarsaw, Poland; ^2^Institute of Psychology, Polish Academy of SciencesWarsaw, Poland; ^3^Faculty of Psychology, University of WarsawWarsaw, Poland; ^4^Laboratory of Psychophysiology, Nencki Institute of Experimental Biology, Polish Academy of ScienceWarsaw, Poland; ^5^Institute of Philosophy, Faculty of Philosophy and Sociology, University of WarsawWarsaw, Poland; ^6^Center for Semiotics, Aarhus UniversityAarhus, Denmark; ^7^Interacting Minds Centre, Aarhus UniversityAarhus, Denmark

**Keywords:** language coordinated interaction, systemic complexity, bias-variance analysis, collective performance, wine tasting and recognition

## Abstract

Most of our perceptions of and engagements with the world are shaped by our immersion in social interactions, cultural traditions, tools and linguistic categories. In this study we experimentally investigate the impact of two types of language-based coordination on the recognition and description of complex sensory stimuli: that of red wine. Participants were asked to taste, remember and successively recognize samples of wines within a larger set in a two-by-two experimental design: (1) either individually or in pairs, and (2) with or without the support of a sommelier card—a cultural linguistic tool designed for wine description. Both effectiveness of recognition and the kinds of errors in the four conditions were analyzed. While our experimental manipulations did not impact recognition accuracy, bias-variance decomposition of error revealed non-trivial differences in how participants solved the task. Pairs generally displayed reduced bias and increased variance compared to individuals, however the variance dropped significantly when they used the sommelier card. The effect of sommelier card reducing the variance was observed only in pairs, individuals did not seem to benefit from the cultural linguistic tool. Analysis of descriptions generated with the aid of sommelier cards shows that pairs were more coherent and discriminative than individuals. The findings are discussed in terms of global properties and dynamics of collective systems when constrained by different types of cultural practices.

## 1. Introduction

Even though we are often not aware of this, our decisions and actions in the world are rarely a solitary enterprise. When going for a job interview, your reaching to take out appropriate clothes seems to be your decision here and now, yet it is constrained by various kinds of cultural contexts. Your choice, an important one, as you are deciding on how much of your own personality you wish to reveal to your future employer, depends on what is acceptable in your culture, on which dress codes have been taught to you by your family (explicitly or by practice), and on the current fashion and how your peers dress on such occasions. You may check the dress code of the company for which you are getting interviewed and check your choices with family members and friends by asking them in person or sending your picture via electronic media.

Doing things together is thus our species' natural mode of being, a fact generally underappreciated in cognitive psychology. Our actions, choices, and decisions practically always have a collective dimension. This togetherness comes in many forms: the presence of others (physical or virtual), engagement of culturally developed artifacts (Hutchins, 1995b; Clark and Chalmers, 1998), or knowledge, how things “ought to be done” or are “usually done,” i.e., the social norms and practices which we acquire from our social surroundings and upbringing (Sidnell and Enfield, 2012; Enfield, 2013; Sinha, 2014).

Before we can start addressing the collective nature of human cognition and behavior we have to be careful in how we define it. From the dynamic perspective we engage, interactions are not just simple combinations of behaviors of two or more individuals. Rather, by entering a social interaction, individuals become parts of a larger systemic organization. New qualities emerge that can only be captured at the collective level. In turn, the emergent level comes to shape individual action and cognition (Schmidt et al., 1990; Hutchins, 1995a; Di Paolo et al., 2008; Schmidt and Richardson, 2008; Riley et al., 2011; Fusaroli et al., 2014). Such systemic level organization of human collectivity arises at multiple timescales: it is effective when engaging each other face-to-face, but crucially depends on being shaped in development (Rączaszek-Leonardi et al., 2013), cultural evolution (Smith et al., 2003; MacWhinney, 2005; Enfield, 2013) and even biological evolution (Lewontin, 2001; Smaldino, 2014). This approach calls for new methods to describe properties of emergent collectivity and link them to the performance and properties of participating individuals. In investigations of movement coordination, concepts such as *coupling* or *functional synergy* have been applied to address aspects of complexity, stability and functional coherence of collective systems (Turvey, 1990; Schmidt and Richardson, 2008; Riley et al., 2011). Considering the collective dimension of systems has brought a focus on the system's level performance. Variables pertaining to the systems as a whole, such as temporal characteristics of their behavior and/or their stability or variability of performance are increasingly often used as indices revealing internal dynamics of such systems (Van Orden et al., 2003). Using such means, one can assess the functional reduction of degrees of freedom that results for a given system from a particular interaction in a particular situation.

Such views on collectivity bring about new perspectives on natural language as it becomes a constitutive element of human interaction. First, language is not considered an individual skill, a categorization tool or a simple vehicle of content. Rather, it is a mean of coordination, enabling and shaping interactions (Halliday, 1977; Schegloff et al., 1996; Rączaszek-Leonardi and Kelso, 2008; Tylén et al., 2010; Raczaszek-Leonardi and Cowley, 2012), which—congruently with the systemic view above—can be operationalized as functional control over the systems' degrees of freedom. Second, the crucial role of language for interaction has to be considered on several timescales (Rączaszek-Leonardi, 2003; Smith et al., 2003; MacWhinney, 2005). These timescales range from on-line processes, when interlocutors dynamically construct linguistic controls appropriate for a current task (Fusaroli et al., 2012; Mills, 2014) to the slower cultural processes of selection and stabilization of linguistic structures and practices useful to control interactions in relevant activities (Rączaszek-Leonardi, 2009). This view carries explanatory potential not only for aspects of emergence of grammar in general but also for the emergence of domain-specific professional argots and even codified linguistic artifacts containing terms and structures selected to enable and facilitate co-action within specific fields of human activity.

This approach to collectivity and the role of language has only quite recently been employed to explain cognitive and linguistic coordination. It charts a field for the study of language in real interactions, over many timescales, utilizing advanced methods for studying complex dynamical systems. Some of the paths in this field are already being empirically explored in a promising way. Recent studies have shown how symbolic constraints can emerge in the course of online interactions (Galantucci, 2005; Fay et al., 2010; Mills, 2014), as well as how they guide the systems' collective task performance (Fowler et al., 2008; Dale et al., 2011; Fusaroli et al., 2012). The synergetic model has proven promising in accounting for the features of on-line communication that best predict performance on simple decision tasks (Fusaroli and Tylén, 2016). However, most studies so far have utilized only simple, one-dimensional tasks, which might have reduced the possible influence of linguistic coordination. Furthermore, questions remain open as to the potential impact of other timescales of language functioning (such as written cultural artifacts). Thus in our study, using the systemic approach sketched above, we aimed to investigate the task-relevant constraining role of language coming from different time-scales in a cooperation involving multidimensional stimuli.

## 2. The study

The present study was designed to assess the impact of two forms of linguistic involvement on the properties of collective systems formed to solve a recognition task. We will address the following questions: First, does spontaneous linguistic interaction affect behavior of a system in a complex perceptual identification and recognition task? Second, is its behavior further influenced by the use of a linguistic artifact, established on a cultural evolution timescale to facilitate communication and performance on the specific task? Third question regards the relation between these linguistic influences (spontaneous talk vs. artifact use) and their possible interaction. The hypotheses are formulated regarding both the performance of the collective systems and the kinds of errors that the systems make, which indirectly testify to the internal dynamics of the systems, which render specific discriminatory and recognition capabilities. For this purpose we rely on the *bias-variance decomposition framework* (Geman et al., 1992; Domingos, 2000). The participants' behaviors will be analyzed in terms of the systemic and multi-scale view introduced above. This means that the object of study will be *the systems* constituted through the use of various types of linguistic coordinators.

Bias-variance decomposition is a tool, which allows to distinguish between bias—systematic error and variance—random, uncontrolled error. This kind of analysis becomes increasingly important if we consider systems making decisions in open, dynamic environments (Gigerenzer and Brighton, 2009). In our case bias and variance can be treated as indices of the internal dynamics of the system. When we consider systems that learn from interaction with the environment, with each system having slightly different experiences (data sample), variance is connected with the sensitivity of the system to individual samples: a system with high variance will produce very complex rules of judgment, tailored to the specific data it has been exposed to; a system with low variance will produce simpler rules ignoring the specific details of individual samples. High variance implies many internal degrees of freedom, which enable the system to fixate on the specific details of the data, but leads to a loss in the ability to generalize. High bias, on the contrary, relates to a low number of internal degrees of freedom, when the system is unable to cope with the problem's complexity, systematically skewing the system's performance in one direction. To gain intuition about these dependencies, we can think about people with various introspective abilities engaging in common social tasks, for example a person making a decision to take the floor during a large gathering, for instance, a scientific conference. People with low introspective abilities will act according to simple rules: for example, whenever their general confidence level is high they will start talking, failing to notice more subtle contexts, which make their action ill-timed (for instance, another person trying to say something). Their actions will be schematic and they will make mistakes in certain situations (low variance, high bias). On the other hand, people with high introspective abilities and a complex model of the situation will be very sensitive to fluctuations of their own mood and subtleties of the circumstances. In many cases they will overcomplicate things by analyzing unimportant details, for instance, they will try to predict the mood of all the people in the audience and if their comment really fits the discussion. Their behavior will be flexible but unpredictable, and sometimes the overwhelming number of details will prevent them from taking any action at all (high variance, low bias). This illustrates a notion of bias-variance tradeoff because for a specific problem complex systems with low bias tend to have higher variance and vice-versa. The same phenomena which govern the behavior of an individual occur also on the collective level, which is the case in our study, where error decomposition is applied to provide insights into how different forms of linguistic constraints influence the description and recognition of complex perceptual stimuli.

## 3. Design and hypotheses

In order to assess how two forms of linguistic collective engagement, i.e., spontaneous conversation and the use of a domain-specific cultural artifact, constrain cognition in a complex recognition situation, we needed a task which would: (i) involve complex, multidimensional stimuli; (ii) be difficult enough to yield sufficient performance variability, (iii) not be widely established in everyday language, but, on the other hand, (iv) have a professional, domain-specific, culturally created argot, codified in a linguistic artifact.

Therefore, we chose a wine tasting and recognition task. While being sufficiently difficult and complex, wine recognition is an intuitive task for most participants, and naturally performed as both a solitary and social activity (Lehrer, 2009). The culture surrounding wine consumption is rich and diverse, and a professional language has been developed for wine description, codified in so-called *sommelier cards*. This professional language is not widely known, nor does it correspond clearly with the lay, everyday language used in the novice's “wine talk” (Solomon, 1990). Additionally, multiple existing studies on wine perception, description, and recognition provide a useful background that can guide the selection of the participants and materials (Solomon, 1990; Hughson and Boakes, 2002; Lehrer, 2009; Zucco et al., 2011; Royet et al., 2013).

In order to operationalize our main research questions in a wine recognition task, we employed a two-by-two factorial design: individual vs. pairs (where the requirement of joint decision elicited spontaneous linguistic interaction); and the presence vs. absence of a cultural artifact for wine description (a sommelier card). Thus the conditions were as follows:

1. Individual tasting and recognition of wines (later referred to as “individual, no card”);
2. Individual tasting and recognition using a sommelier card (“individual, card”);
3. Tasting and recognition by spontaneously communicating pairs (“pair, no card”);
4. Tasting and recognition by pairs using a sommelier card (“pair, card”).

Performance was measured in terms of recognition accuracy (score, error decomposition) and the quality of wine descriptions. Recognition accuracy was measured across all conditions and errors were analyzed in terms of systems' bias and variance. In the sommelier card conditions we were also able to comparatively assess the properties of wine descriptions, as the sommelier card provided a limited set of dimensions to be quantified. We were especially interested in how the descriptions produced by pairs vs. individuals differed in their coherence (i.e., similarity across participants within the same condition) and in their ability to separate the wine samples (i.e., how little overlap there was between the different descriptions of wines).

We predicted that both kinds of collective engagements (interacting in real time with a partner or with the cultural scaffold of a sommelier card) would lead to increased accuracy in wine recognition. For systems relying on spontaneous interaction, we expected such benefits to arise from jointly created linguistic controls (shared vocabulary attuned to the task) that would guide collective attention to relevant dimensions of the taste experience (Fusaroli et al., 2012; Tylén et al., 2013). We also expected benefits from using the sommelier card. The sommelier card is a tool, which embodies years of professional experience, offering precise dimensions along which the stimuli can be organized. Thus, both pairs and individuals with a sommelier card should outperform their counterparts without it, as they can rely on a history of culturally selected dimensions to guide their descriptions and recognition processes. Whether the benefits of the two types of collectivity would be additive or interact was an open question.

Crucially, the bias-variance framework presented above allows for making predictions about the kind of errors characteristic for each system. Since, as explained above, the role of language is to functionally bind the degrees of freedom of a system, we can expect that adding linguistic constraints can lead to a decrease in variability of a system's performance. In particular, we expected that adding functional constraints in the form of a sommelier card would decrease the systems' degrees of freedom along culturally selected dimensions, therefore producing lower variance. Questions pertaining to individual vs. collective use of sommelier cards remain open for now: on the one hand, using spontaneous language should also constrain a system's degrees of freedom; on the other, the presence of another person may impinge on the complexity of a system in a way that could obscure this influence.

Finally, we also expected differences in the quality of descriptions prepared by individuals and pairs using the sommelier cards. Previous studies have shown that descriptions created by novices show little similarity and systematicity (e.g., Solomon, 1990). Bringing collective resources to the task should result in increased coherence (similarity of objects within one class) and discriminativeness (dissimilarity of objects belonging to different classes) of descriptions created by pairs compared to those created individually.

## 4. Materials and methods

### 4.1. Experimental task

The task was to smell and taste three target wines in order to recognize them, after a break, among six wine samples. A pilot study was employed to identify an optimal number of wine samples, which would provide enough performance variability with a minimal amount of alcohol to be imbibed. As a wine sample contained 30 ml of wine, each experimental session (1–1.5 h) involved a maximum amount of 270 ml of wine available for consumption. The invited participants were informed that the study involved alcohol consumption which may influence their driving ability. Participants could measure their blood alcohol level with a breathalyzer. Out of 120 participants 102 had measured their alcohol level and 90 of the readings were 0. At maximum 0.19 per mille alcohol were observed, which is below the limits for drivers in Poland.

### 4.2. Participants: recruitment and demographics

Hundred and twenty three participants (85 females, one participant did not declare a gender) took part in the experiment. Participants' age ranged from 18 to 40 (*M* = 23.01, *SD* = 3.80). The majority of participants were university students or had higher education. Potential participants were contacted mainly through social media. They filled in a questionnaire, checking the following in/exclusion criteria: legal age, contraindication to the consumption of alcohol, smell or taste disorders, professional knowledge about wines, frequency of red wine consumption, and fluency in Polish (The questionnaire is provided in Supplementary Material S1.1.1). Informed by studies on the influence of age on olfaction (Doty, 1989; Hummel et al., 2007), we decided to recruit only participants younger than 50 years. Those who met the criteria were invited to participate.

All participants were wine tasting novices, that is, they had only cursory knowledge related to wine culture and possible ways of describing wines. The reasons for this choice were threefold: first, to avoid possible influence of earlier knowledge, which might be present in wine experts (Zucco et al., 2011); second, to avoid a possible verbal overshadowing effect which, according to some studies (Schooler and Engstler-Schooler, 1990; Melcher and Schooler, 1996; Parr et al., 2002) might occur especially when perceptual skills exceed verbal ones, which has been found especially among intermediately skilled participants, see Melcher and Schooler (1996) and Ryan and Schooler (1998). Third, we wanted to be sure that the nature and quality of the vocabulary would indeed be different in the spontaneous conversation and sommelier card conditions of our study, which in the case of experts could not be assured.

Due to these concerns, data from one participant in the individual condition and from one pair was excluded from the analyses because they informed the experimenter or demonstrated an extensive knowledge about wines. The final number of cases analyzed for each condition was as follows: Individual/no card: 20; Individual/card: 20; Pair/no card: 21; Pair/card: 19.

### 4.3. Wine selection

The wines, both target and filler, in the final wine set were dry, red and had rather similar character. The selection was based on decisions of two professional sommeliers and the results of a pilot study. The aim was to maximize resolution in performance and avoid ceiling effects. This resulted in the following wine list:

- Epicuro Aglianico 2005, Italy, Campania, IGT Beneventano
- Varvaglione Primitivo del Salento 12 e Mezzo 2012, Italy, Puglia (target)
- Le Versant Cabernet Sauvignon 2008, France, Languedoc-Roussillon
- Masseria Trajone Nero d'Avola 2006, Italy, Sicily. (target)
- Altarius Crianza 2010, Spain, Rioja, DOC Rioja (target)
- Cubo Seleccion Tempranillo 2011, Spain, La Mancha

### 4.4. Sommelier card

A sommelier card is a cultural artifact that contains linguistic categories, which are used by professionals to judge the quality and the character of wine. Several such tools are presently used across the world, most notably the Associazione Italiana Sommelier card (Italian), Wine and Spirit Education Trust card (English), Deductive Tasting Format (American), or Feuille d'analyse sensorielle (ASNCAP) (French). In this study we chose a slightly simplified Polish version of the Associazione Italiana Sommelier card, which for several years has been used among the Polish sommeliers. This means that the key dimensions used in the card had the Polish terms agreed upon by the Polish sommeliers and used in professional writing. With the help of two professional sommeliers, we removed items that might be misleading for a non-expert because of meanings diverging from everyday language, and included additional explanations for some terms (such as “persistence” or “tannins”).

The resulting sommelier card consisted of 21 items (scales and questions) pertaining to taste (9 items), smell (10 items) and general characteristics of wine (2 items). A comment section was included, where the participants could make their own descriptions if they felt it would help them make correct recognitions. An English translation of the sommelier card can be found in Supplementary Material S1.2. Both individuals and pairs were given the same card. Pairs shared a sommelier card and gave their joint answer for each item.

### 4.5. Experimental procedure

**Figure 1:**
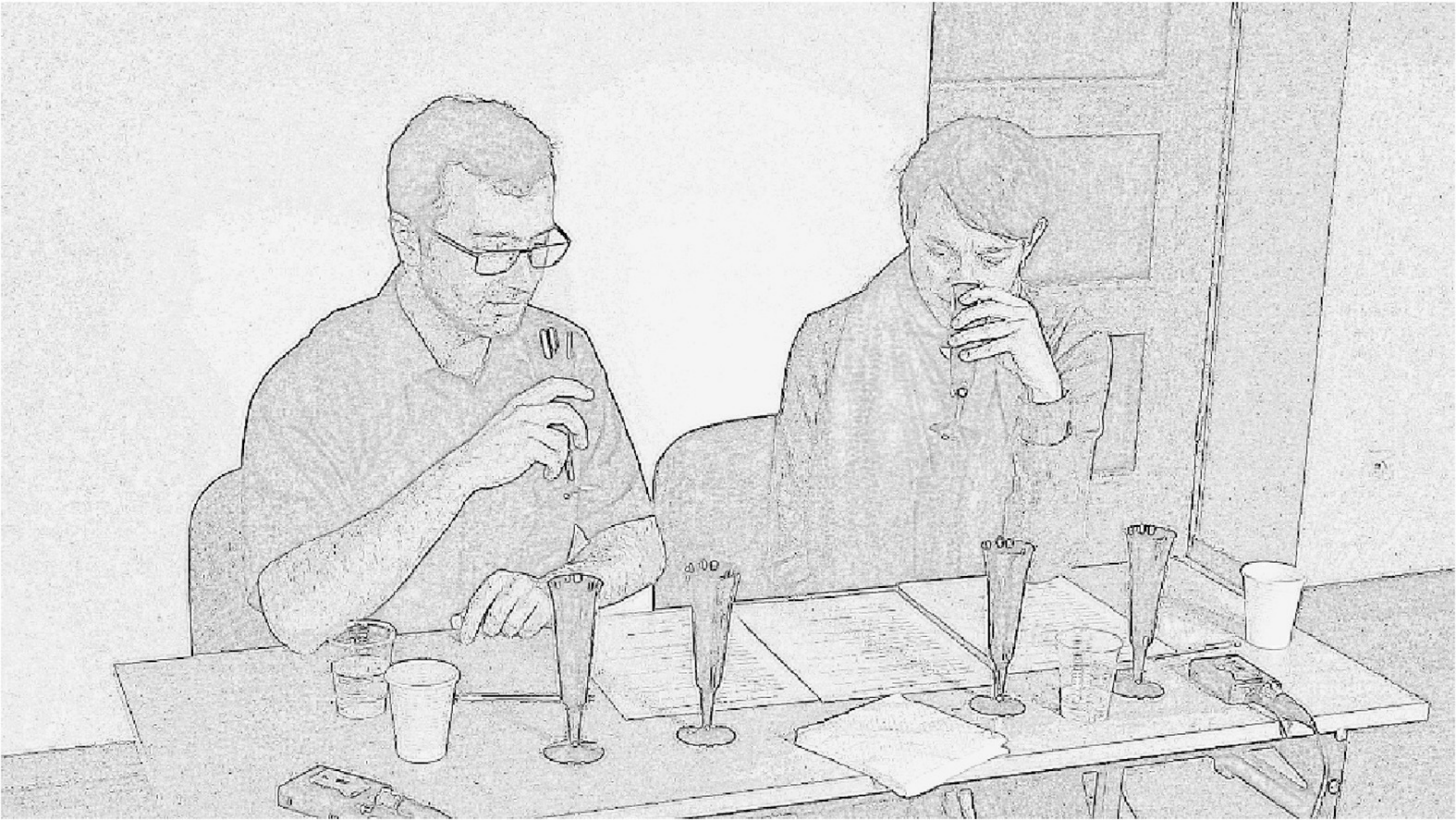
**Participants tasting wines during the learning phase of the experiment**.

The experiment was conducted following the ethical guidelines for psychological research and approved by local ethical committee of Institute of Psychology, Polish Academy of Sciences. Upon arrival, the participants signed informed consent forms and were assigned to one of the experimental conditions. The experimenter explained the task: to taste and smell wines, in order to recognize and identify them later in a larger set of other wines. Each participant was then provided with three samples of the target wines, each labeled with a number: 1, 2, or 3. The labels were consistent between participants and they were informed about this. Figure 1 depicts the experimental setup during the learning phase.

In the sommelier card condition, three sommelier cards were provided, one for each target wine. Participants were instructed to use the sommelier cards for wine descriptions only—numbering or otherwise marking them was not permitted.

To prevent subjects from using additional visual cues, such as wine color or consistence, the samples were presented in black, opaque plastic glasses. Sessions involving pairs were recorded using a video camera and voice recorder. There were no time limitations on the learning phase—participants just signaled the experimenter when ready. The participants would then solve a series of unrelated spatio-visual tasks for approximately 40 min. Subsequently, participants were given six wines (the three targets plus three distractors), labeled with capital Latin letters A–F. Participants had to place correct numbers on three out of six presented wines. In the pair condition the participants were to provide a joint answer. No time limit was imposed in any of the conditions.

After completing the experiment, the participants filled a short survey querying their age, gender, whether they were smokers, the perceived difficulty of the task, and the perceived tastiness of each wine. In the pair condition, the questionnaire contained additional items assessing the relatedness of pair members and the evaluation of the level of cooperation during the session.

The quantity of the wine left was measured. Finally, participants could measure their blood alcohol level with a breathalyzer.

## 5. Results

Raw data from the experiment is included as Supplementary Table S2. Analyses were conducted on three levels: (1) recognition accuracy in four experimental conditions, (2) condition specific patterns of bias and variance, and (3) analyses of the discriminativeness and coherence of the sommelier cards filled by individuals and pairs. Additional analyses assessed the character of the information integration resulting in pairs' wine descriptions. Finally, we provide preliminary data on quantitative aspects of verbal interactions that may have influenced performance.

### 5.1. Recognition accuracy

Task performance was measured as the number of wines accurately labeled by participants (“identification score”). First, we assessed whether participants performed above chance in the four conditions. Since the wines are chosen simultaneously, not sequentially, calculating the baseline random performance is not trivial—for the description and mathematical formulas see Supplementary Material S1.1.2.

Table 1 presents probabilities of obtaining a given score by chance. Observed distributions of scores in specific conditions are given by Table 2. We applied goodness of fit test with simulated *p*-values (5000 samples) to assess if the scores differ from the random baseline. As can be seen, identification scores in all conditions are very unlikely to be obtained by chance.

**Table 1:** **Probabilities of obtaining particular score value by chance under random performance**.

	**0**	**1**	**2**	**3**
Identification score	0.592	0.325	0.075	0.0008

**Table 2:** **Frequencies of wine identification scores, tabulated by condition (***N*** = 80)**.

	**0/3**	**1/3**	**2/3**	**3/3**	***N***	***M***	***SD***	***p*-value**
Individual, no card	10	6	1	3	20	0.85	1.09	0.0014
Pair, no card	10	4	5	2	21	0.95	1.07	0.0014
Individual, card	8	5	6	1	20	1.00	0.97	0.0110
Pair, card	1	12	5	1	19	1.32	0.67	0.0010

*The first four columns represent counts of particular scores, e.g., “2/3” means two wines out of three were identified correctly*.

In order to compare performance in the four experimental conditions, we performed a modified rank-based Brown-Forsyth test for variance inequality, which is median-based equivalent of Levene's test, more robust in case of non-normal distributions. Obtained *p*-value 0.0216 means that variances among groups differ significantly. Because of unequal variances and discrete score values distributed non-normally, to assess central tendencies we used Kruskal-Wallis test instead of standard ANOVA. The analysis yielded no significant results (*p*-value = 0.2328).

**Figure 2:**
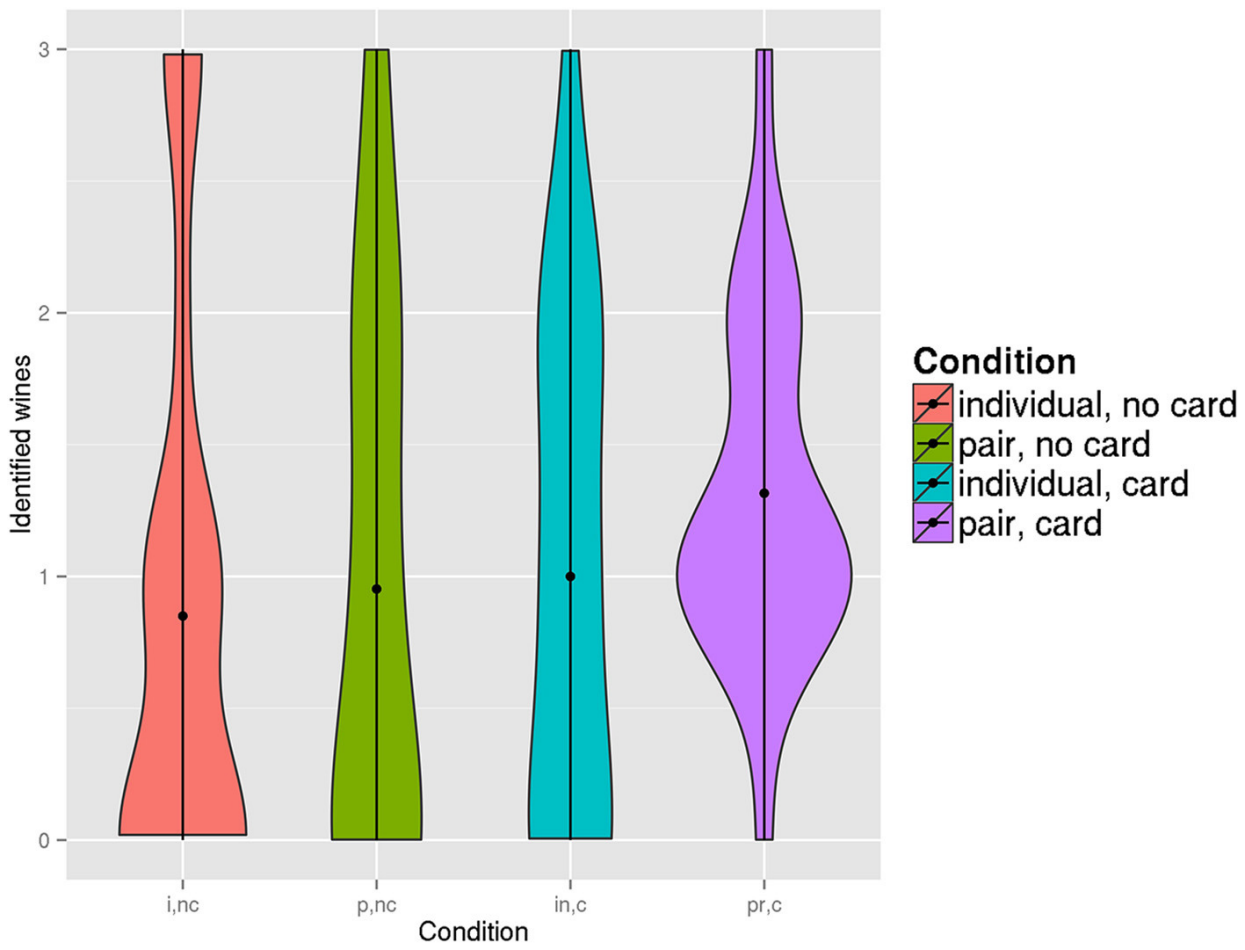
**Number of wines recognized and placed correctly in each condition**.

It is important to notice that even though the average scores in the 4 conditions do not differ, there is a significant difference in the overall scores distribution (Figure 2). For the conditions with sommelier cards, especially for pairs with sommelier card, distribution of scores gravitates toward the middle. Those differences between conditions were found significant by Fisher's exact test comparing numbers of medium scores (1 or 2 correct recognitions) and numbers of extreme scores (0 or 3) (*p* = 0.0002).

### 5.2. Bias-variance analysis

In the previous section we evidenced important differences in score distributions. To gain insight into the nature of errors, we used the bias-variance decomposition (see Introduction) analyzing placements of individual wines instead of aggregated scores. This allows for the analysis of distinct patterns of error structure in more detail. The procedure treats each system as a classifier in a supervised classification task (i.e., a task in which correct labels are given in the learning phase and in the recognition phase the classifier is expected to reconstruct the correct labeling). Error of the classifier can be attributed to three sources: bias, variance and noise.

We treat systems from each of experimental conditions as a classifier population and identification of each wine sample as a single instance of a learning problem. Error decomposition is performed for each population separately, which allows a meaningful comparison between conditions. In this context, bias is a systematic tendency of the systems within a specific condition to confuse two specific wines (answers are systematically skewed), while variance is the diversity of their answers (answers are more random). To define these concepts in a quantitative way, we apply the bias-variance decomposition schema proposed by Domingos (2000). The decomposition has the following form:

$$\begin{array}{l} E\left(x\right)=c_{1}\left(x\right)N\left(x\right)+B\left(x\right)\;+\;c_{2}\left(x\right)V\left(x\right) \end{array}$$

where *E*(*x*) is the expected error that the classifier makes on sample *x*, *N*(*x*) is noise, *B*(*x*) is bias, *V*(*x*) is variance and *c*_1_(*x*) and *c*_2_(*x*) are special coefficients dependent on the sample *x*.

Specific components of error are estimated by averaging over all systems and all samples within each condition. We calculate bias, variance and error for each of the three wines recognized by the participants and average the results. Let's denote y^*^ as the correct class for a given sample, *y* as the class predicted by an individual system and *y*_*m*_ as the class most often predicted among all the systems. In this context we assume noise *N* = 0, that is, wine labels represent the true state of the nature. The overall error *E* is calculated as the fraction of samples identified incorrectly which is an estimation of *P*(*y* ≠ y^*^). Bias *B* is the error of the main prediction, i.e., a classification based on the majority vote of all systems in the specific condition: $P\left(y_{m}\neq y^{*}\right)$. Variance *V* is the fraction of answers different from the dominant answer: *P*(*y* ≠ *y*_*m*_). Coefficient *c*_2_ is a function of sample *x*. For each sample for which the main prediction is correct (hence bias = 0), *c*_2_ is equal to 1. Otherwise, $c_{2}=-P\left(y=y^{*}\wedge ym\neq y^{*}\right)$ i.e., it is proportional to the probability of choosing the correct answer due to variance. This means that for a biased classifier variance may actually reduce the error, because it creates an opportunity to predict a label different from the main prediction.

It should be noted that bias and variance estimation is approximate in these experimental data because of (a) small number of samples (3) identified by each system, (b) the fact that the tasks of identifying wines 1, 2, 3 were not really independent. However, even this imperfect estimation allows for a meaningful comparison of different experimental conditions.

Results of the bias-variance decomposition for each condition are presented in Table 3. In comparison with individuals without sommelier card, pairs without sommelier card have a smaller bias and slightly larger variance, which results in error on roughly the same level. Pairs with sommelier cards, on the other hand, have a larger bias than pairs without card but much smaller variance. The reduced variance of answers was also visible as reduced variance of scores in previous analyses (see Figure 2). Individuals with sommelier cards have a slightly smaller error than individuals without cards, but the difference (due to a slight decrease of variance) is so small that we can say that individuals were mostly unaffected by the use of somelier cards.

**Table 3:** **Error, bias and variance in the four experimental conditions**.

**Condition**	**Error**	**Bias**	**Variance**
Individual, no card	0.71	0.67	0.63
Pair, no card	0.68	0.17	0.68
Individual, card	0.67	0.67	0.62
Pair, card	0.56	0.33	0.54

To calculate statistical significance of the differences we performed a permutation test: we repeatedly (2000 times) randomly split the data into two groups and counted how many times more extreme values of bias and variance were produced. The results presented in Table 4 suggest that the presence of a sommelier card in pair condition significantly alters bias and variance composition, as it is systematically different from all other conditions. In individuals, the sommelier card does not seem to have any influence. These findings motivate a more in depth analysis of the sommelier card-assisted descriptions produced by pairs as compared to those produced by individuals.

**Table 4:** **Results of permutation test comparing bias-variance decomposition between different conditions**.

	***p*-value**
Individual, no card vs. pair, no card	0.1295
Individual, no card vs. individual, card	0.2430
Individual, no card vs. pair, card	0.0370
Pair, no card vs. pair, card	0.0470
Individual, card vs. pair, card	0.0495

### 5.3. Analysis of descriptions through sommelier cards

The analysis above show that sommelier cards affected the performance of pairs to a greater degree than performance of individuals. Therefore, the question arises if we can see this difference also on the collective level through the quality of sets of descriptions produced via sommelier cards by individuals and pairs.

To answer this question we compared coherence and discriminativeness of descriptions prepared by pairs with those prepared by individuals. We had 19 pairs and 20 individuals each filling three sommelier cards, resulting in 57 cards filled in by pairs and 60 cards filled in by individuals. The 21 items from the sommelier card were encoded as a 21-dimensional vector. Since the number of options in each item varied—from two to five—we performed rank normalization: for each item its values were replaced by their ranks in the set of all sommelier cards. This procedure guaranteed that all of the items contributed to the analysis equally, regardless of the number of levels.

As a measure of coherence we used a silhouette score (Rousseeuw, 1987). It is based on the idea that an informative set of descriptions of the same wine should be more similar, while samples of different wines should be as distinct as possible. Formally, for each sample the silhouette score is a relation of its mean distance from points belonging to its class and its mean distance from the points of the closest foreign class. More formally: *s* = (*b*−*a*)/*max*(*a, b*), where *a*—mean intra-class distance, *b*—minimal mean inter-class distance. Silhouette scores look at each sample individually and the mean silhouette score value may be seen as a measure of coherence of the set of descriptions.

In order to measure the descriptions' discriminativeness, i.e., their usefulness for discriminating different wines, we employed multinomial logistic regression. The independent variables were the 21 sommelier card items, the dependent variable was “wine label,” and the model's accuracy in reclassification scenario was used as a score—the higher the accuracy, the more discriminative the description set. Note that the regression model was not used for inference, but rather as a measure of linear separability.

**Figure 3:**
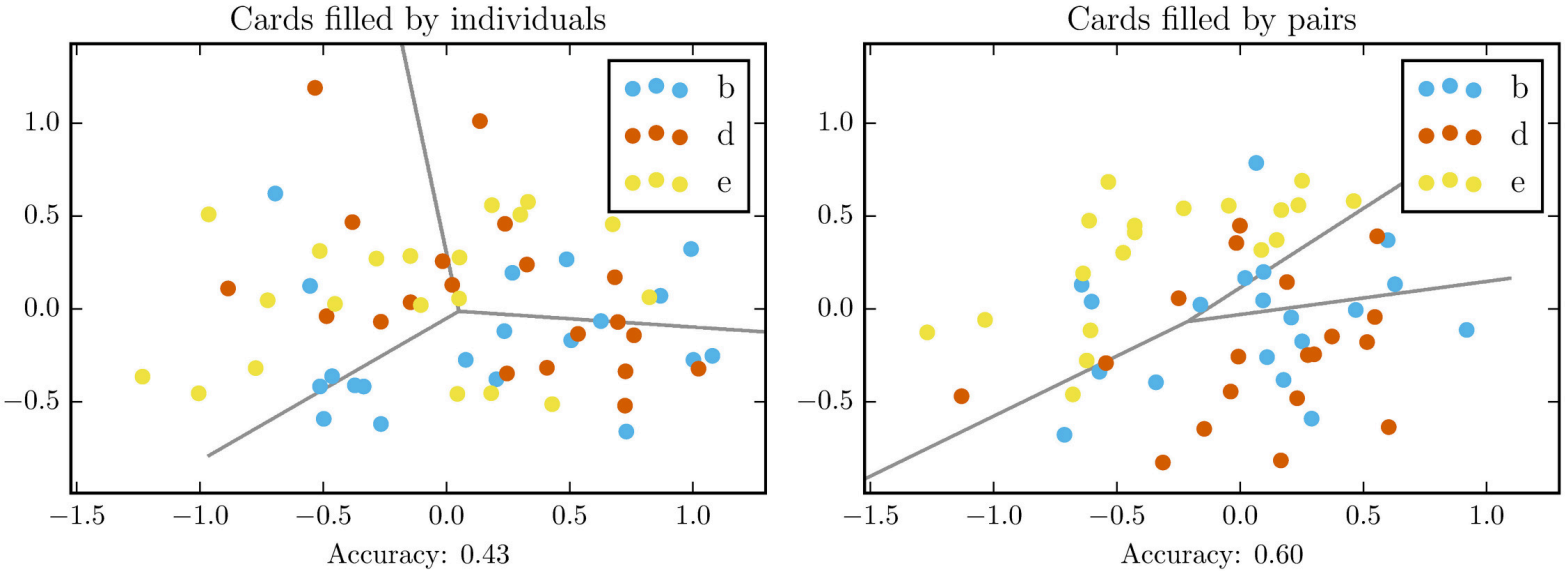
**Dispersion of filled sommelier cards after rank normalization and transformation with PCA (Principal Component Analysis)**. Gray lines denote logistic regression decision boundary, the model accuracy is reported below.

Figure 3 shows dispersion of wine descriptions after rank normalization and dimensionality reduction through PCA. We applied a simple multinomial logistic regression model to look for regularities in the data. The independent variables were top two principal components, the dependent variable was either wine label or experimental condition. We observe a clear difference between descriptions of individuals and pairs (accuracy 0.43 vs. accuracy 0.6), which means that those prepared by pairs are more discriminative.

To obtain more meaningful results we compared differences between the two groups (individuals and pairs) in the original 21-dimensional space. We tested two hypotheses: (1) that the scores in each group are different than obtained by chance and (2) that the scores between the two groups (pairs and individuals) differ on those measures. Since such design is beyond the assumptions of standard statistical tests for linear models, significance of the obtained results was verified using permutation tests with 2000 permutations, conducted independently for each measure.

First, we compared the obtained results with the random baseline for individuals and pairs separately. Class labels of the descriptions were permuted randomly and the number of times when the permuted set outperformed the original one was counted. Results are presented in Tables 5-I,II. Pairs performed significantly better than random, while individual descriptions are on the baseline level. This means that, according to our criteria, on the population level the information content of individual descriptions is close to none.

**Table 5:** **Comparison of coherence and discriminativeness of descriptions prepared by individuals and by pairs**.

		**Silhouette score**	**Logit score**
I	Individual	−0.02	0.57
	Individual (randomized)	−0.02	0.65
	*p*-value	0.61	0.93
II	Pair	0.01	0.81
	Pair (randomized)	−0.02	0.66
	*p*-value	0.0005	0.0005
III	Pair	0.01	0.81
	Individual	−0.02	0.57
	*p*-value	0.0005	0.0005
IV	Pair	0.00	0.88
	Synthetic pair	−0.02	0.76
	*p*-value	0.02	0.07

*P-values for various dispersion metrics obtained through group split permutation test. Data after rank normalization*.

The next step was to compare pairs and individuals directly. In each split of the permutation test we divided all the systems into two groups randomly. Then we calculated the value of each measure for each group. We counted the number of times when the obtained values were more diverse than those found between pairs and individuals. *P*-values returned by the described test are reported in Table 5-III. Significant differences were obtained both for silhouette scores and logistic regression reclassification accuracy. This suggests that descriptions made by pairs were both more coherent and more distinctive, allowing for a better classification than descriptions made by individuals.

An additional analysis was performed in order to gain more insight on how the information integration process occurred in pairs. One of the simplest possible mechanisms for the participants would be filling out the sommelier cards individually and then averaging the answers to obtain a pair decision. To test whether the participants could have employed such a procedure, we constructed artificial data points by randomly pairing and averaging points corresponding to the sommelier cards filled by individuals. We performed a permutation test comparing such synthetic sommelier cards with cards prepared by the real pairs. We randomly paired individual experiment participants to construct 10 × 3 synthetic sommelier cards and compared them with 10 × 3 sommelier cards produced by 10 randomly selected individuals/pairs. We compared the scores for both groups and counted the number of cases when the score obtained for synthetic pairs was larger than the score obtained for real pairs. The experiment was repeated 2000 times. *P*-values returned by the described tests are reported in Table 5-IV. Data from the real pairs were significantly more coherent than the synthetic data (*p* = 0.02), while the difference in discriminativeness was on a tendency level (*p* = 0.07). These results demonstrate that the mechanism of information aggregation employed by the participants who collectively filled in sommelier cards is more complex than simple averaging. Through the conversation they were able to effectively combine information from their senses and their understanding of sommelier card categories to improve the quality of their descriptions.

### 5.4. Analysis of verbal interactions

In addition to performance data and analyses of the sommelier cards, we transcribed the video recordings, which allowed for quantitative characterization of pairs' verbal exchanges. We manually annotated the transcripts by assigning predesignated categories to words and phrases according to their communicative function. This, in turn, allows us to investigate more semantic and pragmatic aspects of pairs' collaborative exchanges. The most important category used in our present analyses was the “descriptor” category, which contained all vocabulary items used to describe properties of specific wines (taste, smell, etc.). The details of the transcription procedure and the full list of categories can be found in the Supplementary Material S1.1.3. Below we present preliminary observations from the analyses of the transcripts, to investigate which properties of the linguistic interactions are systematically related to pairs' performance.

In order to determine whether performance could be explained by the volume of verbal exchange, we examined the number of phrases and words used in the conversations (referred to as phrase count and word count), as well as the duration of the conversation. For each of these measures two Kendall's rank correlation (tau) tests were performed, separately for the card and no-card condition. No significant relationship was found for any of these measures.

As a measure of conciseness of conversation we used the mean of the logarithm of the length of utterance (short: MLU). We calculated lengths of uninterrupted utterances by a single speaker. Conciseness of conversation can be seen as an indicator of more efficient communication and language use—as less talk is required to convey the information on a single turn (Wilkes-Gibbs and Clark, 1992; Clark, 1996). Hence, we can expect that MLU would correlate negatively with performance. The Kendall's rank correlation test revealed a significant negative correlation between MLU and identification score in the sommelier card condition (*r*_τ_ = −0.38, *p* = 0.039). For the no-card condition the correlation was not significant (*r*_τ_ = −0.29, *p* = 0.098). The difference in MLU between card and no-card conditions was not significant according to *t*-test (*t* = 1.88, *p* = 0.07). These analyses suggest, that it was not the quantity of talk that influences performance, but rather the qualitative aspect of the exchange.

Next, we analyzed the vocabulary used to describe wine properties by experiment participants. From earlier work (Fusaroli et al., 2012; Fusaroli and Tylén, 2016) we expected to see a certain “homing in on” important dimensions for the particular task in more successful pairs when compared to those that were less successful. We therefore analyzed the elements of transcripts that were classified into the “descriptor” category. We expected the consistent use of wine-related vocabulary to be correlated with recognition performance and that more consistency will be displayed by pairs with sommelier cards. To measure the vocabulary consistency we introduced three measures:

- Type to token ratio (TTR)—ratio of unique words to all words in “descriptor” category. Smaller TTR means more concise vocabulary.
- Common vocabulary between phases (CVP)—ratio of unique words occurring in both phases (learning and recognition) of the experiment to all unique words in “descriptor” category. Larger CVP means more consistent vocabulary.
- Common vocabulary between speakers (CVS)—ratio of unique words used by both speakers to all unique words in “descriptor” category. Larger CVS means that more descriptors are shared.

Differences of those measures between experimental conditions are given by Table 6, and correlation with performance by Table 7. We can see that for card condition vocabulary is more concise and consistent (significant effects for TTR and CVS), and that consistency correlates positively with performance only in no-card condition (significant effects for CVP and CVS).

**Table 6:** **Results of ***t***-tests comparing vocabulary consistency measures between sommelier card and no sommelier card experimental conditions**.

**Measure**	**No-card**	**Card**	***t***	***p*-value**
TTR	0.44	0.28	3.32	0.003
CVP	0.12	0.19	−1.59	0.12
CVS	0.18	0.25	−2.35	0.02

**Table 7:** **Results of Kendall's rank correlation for vocabulary consistency measures and performance in sommelier card and no sommelier card experimental conditions**.

**Measure**	**Condition**	***r*_τ_**	***p*-value**
TTR	No-card	−0.26	0.14
	Card	−0.06	0.73
CVP	No-card	0.40	0.03
	Card	0.02	0.91
CVS	No-card	0.35	0.01
	Card	−0.14	0.26

The results of these analyses suggest that consistency in description is important for the wine recognition task. Pairs with sommelier cards used more consistent vocabulary, and displayed similar characteristics as the most successful pairs without cards. The correlations in card condition were probably not visible since the consistency was already very high (in a sense, “forced” by the card).

## 6. Discussion

In this paper we aimed to use a systemic approach to investigate the task-relevant constraining role of language coming from different time-scales. The timescales involved in our experiment included the biological level, connected with innate perceptual capabilities of individuals, the cultural level, including established categories in wine language, individual experience with similar types of stimuli (wine), and finally the time scale of real-time events consisting of the learning phase and the recognition phase. We controlled biological, cultural and individual experience scales to some degree through our recruitment procedure. Effects in the recognition phase were observed as the systems' performance, analyzed both as mean error and through bias-variance error decomposition. Additionally, we obtained insights into the learning phase by analyzing descriptions prepared using the sommelier cards. Types of collectivity on here-and-now scale included individual condition (no additional information) and a pair condition featuring spontaneous communication between participants (information integration in a pair). We also introduced a cultural-level constraint on colectivity through the use of an external artifact (sommelier card). We investigated different aspects of memory and decision making by experimentally manipulating these two central factors (collectivity and timescales).

Our analyses revealed that the levels of the first factor, representing different types of collectivity, have little impact on averaged performance scores in the recognition phase. However, it still has a significant impact on the systems' properties as evidenced by condition-specific patterns of error, revealed through bias-variance decomposition.

It occurred that the pair condition did not reduce the overall error. The variance of pairs without sommelier card was not smaller than the variance of individuals (i.e., spontaneous conversation did not seem to constrain the system). If the participants tried to solve the problem independently and then reported the average of their answers, the variance should decrease [according to Bienaymé formula, variance of the mean of uncorrelated variables with the same variance is that variance divided by the number of variables (Hoey and Goetschalckx, 2010)]. This suggests that participants chose a different strategy and made an attempt to adapt and complement each other. The lack of decrease in variance indicates that the language in spontaneous communication did not provide significant constraints. It is possible that the participants were able to influence each other but had difficulties communicating the precise meaning having no experience in wine talk and wine language. Benefits from communication have been argued to occur only when pairs are able to use locally aligned terms that are relevant for a given task (for example, Fusaroli et al., 2012 showed how pairs through verbal interaction calibrated their individual levels of confidence to inform joint decisions) and when the created conceptualizations are consistent during the whole performance. Perhaps in the case of pairs without the sommelier cards dealing with very complex stimuli, the time of the session was too limited for common dimensions to emerge and what we see is the “scouting” phase for useful terms. Indeed, our analyses of recording transcripts revealed that pairs without sommelier cards were using less consistent vocabulary than pairs with cards. Among pairs without cards those which managed to establish some consistency and sharing of the vocabulary were more successful.

Pairs with sommelier cards were characterized by the lowest variance, and bias only slightly larger than pairs without cards. Thus, the lower variance was likely due to useful constraints provided by sommelier card's linguistic categories, reducing the number of degrees of freedom of the system. By organizing their communication around these categories pairs were able to share their insights more reliably and precisely. Such results in collective decision making have been shown in earlier research for less complex tasks (e.g., Bahrami et al., 2010; Denkiewicz et al., 2013) and theoretical models have been developed to test which method of information integration have been used such as, e.g., weighted confidence sharing (Sorkin et al., 2001; Bahrami et al., 2010). It is possible that the present task requires more complex information integration models. This matter requires further investigation.

In the individual condition, the sommelier card provided slight constraints reducing the variance and the overall error, however this effect was very small and was not verified as significant. Thus, the external language categories had a large impact on pairs constraining their communication, but did not influence individuals, who did not have to share their experiences to jointly produce a description.

Further analyses revealed that the constraining effects of collectivity were already detectable in the learning phase. Analyses of the individuals' descriptions coherence and discriminativeness revealed them to be indistinguishable from a random baseline, which means they were not able to use this cultural tool effectively. Sommelier card-assisted descriptions produced by pairs were both more coherent and more discriminative than the descriptions produced by individuals. Importantly, informative sommelier cards cannot be produced simply by averaging scores from non-interacting individuals, thus by-passing true social interaction. This finding indicates that collective benefit effects are contingent on genuine dialogical interaction dynamics (Bahrami et al., 2010; Denkiewicz et al., 2013).

In the introduction, we left it open if the two target factors ‘collectivity’ and ‘engagement of a cultural artifact’ would affect the behavior of the participants in an purely additive or interactive manner. Our data suggests that the influence is interactive rather than additive. Individuals did not seem to benefit from the sommelier cards and the descriptions through cards prepared by them were not as discriminative as descriptions prepared by pairs. This observation suggests that rather than an external memory aid, we should consider the sommelier card as a linguistic tool beneficially constraining communication. The numbers of degrees of freedom cannot be reduced solely by means of using professional verbal categories—meanings of those categories have to be negotiated and clarified in interaction. While analyses suggest that the benefit of pairs with sommelier cards is contingent upon interaction, it remains open whether such effects are due to the co-creation of a shared description vocabulary. This will be subject of further analysis of already gathered transcript data.

Interestingly, the clear significant collective benefit for the quality of descriptions resulted in only a slight increase in performance, which did not reach significance. The framework we used in this paper gives a useful tool to gain insights also into intraindividual process of information integration: taking into account different modalities within an individual can also be interpreted in terms of a “collective” system. Inclusion of multiple modalities links smoothly with research on the so called verbal overshadowing effect. The verbal overshadowing effect, although replicated by many (Schooler and Engstler-Schooler, 1990; Schooler et al., 1996, 1997; Dodson et al., 1997; Finger and Pezdek, 1999), by others is considered controversial (Yu and Geiselman, 1993; Meissner and Brigham, 2001; Memon and Rose, 2002; Memon et al., 2003). It has been shown that intermediate level individuals (non-novices and non-experts) who formulate detailed verbal descriptions of complex non-verbal stimuli experience detrimental effects on recognition in comparison with those who did not formulate such descriptions (Melcher and Schooler, 1996; Ryan and Schooler, 1998). By inviting naive participants, we tried to minimize the possible influence of this effect, however the combined result of better quality of descriptions for the pairs (good verbal coordination) with lack of increase in performance points to this factor as one of possible causes.

Future work should investigate overshadowing effect using bias-variance decomposition framework. This should give a clear answer whether intermediate individuals formulating verbal descriptions suffer from increased bias or increased variance. In this case increased bias would mean that verbal categories indeed overshadow (constrain too strongly) the ability to sensorily distinguish samples. Increased variance, on the other hand, would mean that sensory and verbal categories add up resulting in too many degrees of freedom. This opens possibilities for future research.

In this work we performed some basic analyses of the communication transcripts in terms of the amount of verbal exchange and vocabulary consistency. In the future we are also planning to look deeper into communication transcripts of particular pairs to find insights into specific factors, which constitute successful communication. For example we plan to check how the dynamics of the conversation unfold, and if better performance is linked to the linguistic alignment on key terms over time.

## 7. Conclusions

Our study showed an impact of linguistic interaction in a complex recognition task, although performance benefit that stems from such interaction is not conclusive. Analyzed on the systemic level, this impact can be understood in terms of the kinds of error that various types of collective systems are prone to, which in turn are indicative of the number of degrees of freedom of a system performing the task. In this particular scenario unconstrained communication between members of a pair did not constrain the system, while adding a sommelier card to the pairs' task beneficially reduced the system's degrees of freedom. Thus, we have demonstrated how constraints from different types of linguistic interaction (spontaneous vs. utilizing a cultural artifact created on a slower timescale) influence the system differently.

It is important to note that the effects obtained pertain to the systemic level of the linguistically mediated interactions which were created in our study. Analysis on this level, with the use of methods such as bias-variance decomposition can be informative and helpful as a source of hypotheses about the individual-level cognitive processes that are present in such tasks. Linking these levels of explanation (individual and collective) is crucial for understanding how language functions as a social coordination tool.

## Author contributions

JZ performed the experiments, analyzed the data, wrote the paper, reviewed drafts of the paper. MD performed the experiments, analyzed the data, wrote the paper. AD, AR, JK, PL, MS, AK, ES, KK performed the experiments, wrote the paper. RF, KT conceived and designed the experiments, reviewed drafts of the paper. JR conceived and designed the experiments, performed the experiments, wrote the paper, reviewed drafts of the paper.

## Funding

This work has been funded by European Science Foundation EuroCORES EuroUnderstanding grant DRUST 888/N-EuroUnder/2011/0 to the last author. JZ received funding from Operational Programme Human Capital “Information technologies: Research and their interdisciplinary applications” agreement no UDA-POKL.04.01.01-00-051/10-00 under the European Social Fund and Polish National Science Centre grant number 2015/16/T/ST6/00493. RF and KT were funded by Interacting Minds Centre, Aarhus University. The funders had no role in study design, data collection and analysis, decision to publish, or preparation of the manuscript.

### Conflict of interest statement

The authors declare that the research was conducted in the absence of any commercial or financial relationships that could be construed as a potential conflict of interest.
