# Supplementary material for: Performance of Language-Coordinated Collective Systems: A Study of Wine Recognition and Description
Source: Front Psychol. 2016 Sep 27;7:1321. doi: 10.3389/fpsyg.2016.01321 (PMC5037268; doi:10.3389/fpsyg.2016.01321)
Supplement: Supplementary file 1 [file Presentation1.pdf]

Performance of language-coordinated collective systems: A study of wine recognition and description

Julian Zubek [1,2], Michał Denkiewicz [3], Agnieszka Dębska [4,5], Alicja Radkowska [4], Joanna Komorowska-Mach [6], Piotr Litwin [4], Magdalena Stępień [4], Adrianna Kucińska [4], Ewa Sitarska [4], Krystyna Komorowska [4], Riccardo Fusaroli [7,8], Kristian Tylén [7], and Joanna Rączaszek-Leonardi [3,*]

*Correspondence:
Joanna Rączaszek-Leonardi, ul. Jaracza 1, 00-378 Warsaw, Poland
jraczaszek@psych.pan.pl

1 SUPPLEMENTARY DATA

1.1 Initial questionnaire

1. Are there any reasons you do not want to or can not drink alcohol?
   - yes
   - no

   If the respondent had chosen yes no further questions were asked.
2. Do you have any smell or taste disorders?
   - yes
   - no

   If the respondent had chosen yes no further questions were asked.
3. From the list below choose two alcohol drinks you like the most:
   - beer
   - white wine
   - red wine
   - drinks
   - vodka
   - whisky

   1 point for red wine
4. How would you state your knowledge about wines?
   - very poor
   - rather poor
   - difficult to say
   - rather good
   - very good

5. How often do you drink wine?
   - Less than once a month
   - More often than once a month (how often?).

   1 point for more than once a week
6. What are the criteria you will follow buying a bottle of red wine?
   - look of the label
   - country of origin
   - region of origin
   - wine grapes (What grapes varieties do you know?)
   - sweet/dry

   1 point for region of origin 1 point for wine grapes

A respondent was invited to take part in the experiment if he or she answered very poor, poor or difficult to say for question 4 and scored two or less points for the control questions (3, 5, 6).

1.2 Random performance

The participant gets M wine samples in the first part. In the second part he gets N samples ($N > M$). His task is to identify correctly M samples that occurred in the first part out of N samples from the second part. By recognition we mean stating correctly that the sample occurred in the first phase. By identification we mean identifying the correct wine from the first phase. Number of all possible combinations:

$$\binom{N}{M} M!$$

This corresponds to choosing M samples out of N and permuting them. To calculate the number of combinations such that exactly k samples where recognized correctly, we can imagine the following procedure: first, we choose which k samples out of M will be recognized. Then from the $N - M$ samples which did not occur in the first part we choose the remaining $M - k$ samples. The number of combinations is expressed as:

$$\binom{M}{k}\binom{N-M}{M-k}$$

Then, the number of combinations such that k samples where correctly recognized, and among them l samples where identified correctly is:

$$\binom{M}{k}\binom{N-M}{M-k} D_m(M,k,l)$$

where $D_m(M, k, l)$ is the number of permutations of an M-element sequence such that among k distinguished elements precisely l are on the right place. We want to calculate $D_m(M, k, l)$. We know that M wines was chosen and has to be permuted. k wines were chosen correctly, so now we have to choose among them l wine which will be left on their places (not permuted). The remaining $k - l$ wines have be permuted. What about the $M - k$ wines which was chosen incorrectly? There is no correct placement of them so they can be either permuted or unpermuted. We consider all the possibilities, namely that

$0, 1, \ldots, M-k$ out of $M-k$ wines were left on their places. The formula looks as follows:

$$D_m(M,k,l) = \binom{k}{l} \sum_{0}^{M-k} \binom{M-k}{i} D(M-l-i,0)$$

where $D(M-l-i,0)$ is the number of permutations of $M-l-i$ elements such that not a single element is left on its place. Such number are called rencontres numbers and can be calculated which the following formula:

$$D(n,0) = \left[\frac{n!}{e}\right]$$

In the case of our experiment there were $M = 3$ wines in the first part and $N = 6$ wines in the second part. The probabilities are calculated by dividing number of combinations for specific k and l by the number of all combinations.

$\frac{l}{k}$	0	1	2	3
0	0.050			
1	0.300	0.150		
2	0.225	0.150	0.075	
3	0.017	0.025	0.000	0.008

Random chance of recognizing k wines and identifying l wines in an experiment with $M = 3$ wines in the first part and $N = 6$ wines in the second part.

1.3 Details of transcription procedure

40 conversations were recorded in 2 conditions: spontaneous conversation during joint task performance and conversation while filling a sommelier card for each wine sample. All conversation were in Polish.

Transcriptions of conversations in learning and recognition phase were made using Aegisub program, that was originally designed to create and modify subtitles. Aegisub allows to create timed text and display lines within exact time frame. We provided transcripts of 14,7 h of records (2 records x 41 pairs). A single uninterrupted phrase by a single speaker corresponding to specific time frame was dubbed “utterance”. Segmentation of speech into utterances followed natural flow of the conversation and was generally unambiguous.

We used PANTERA Morphosyntactic Tagger (`https://github.com/accek/pantera-tagger`) to perform lemmatization of the transcribed text, reducing all vocabulary to its base (uninflected) form.

Then the transcripts were manually annotated by assigning words to various categories according to their communicative function. Categories were devised in order to explain the dynamics of the conversation as well as possible. The categories were:

- Descriptor – vocabulary item used to describe properties of specific wine (taste, smell, etc.), e.g. “dry”, “pungent”.
- Wine number – reference to wine number, e.g. “wine number one”, “the second wine”.
- Wine letter – A, B, C, D, E, F.

- Wine reference – any other reference to specific wine deducible from the context, e.g. “this”, “that”, “every”, “none”, “previous”, “all”.
- Evaluator – vocabulary item describing ones attitude to the wine, e.g. “good”, “tasty”, “like”, “favorite”.
- Comparison – vocabulary item used to compare wines, e.g. “better”, “than”, “too”, “as”.
- Subjectivisator – vocabulary item used to express ones feeling or individual experience, e.g. “I think”, “I”.
- Reference to partner – vocabulary item referring to the feeling or individual experience of the partner, e.g. “What do you think?”, “What would you say?”, “And you?”.
- Certainty – vocabulary item used to describe the degree of certainty, e.g. “maybe”, “sure”, “rather”, “seem”, “I do”.

Seven independent coders were trained to assign the words to the categories described. Some conversations were transcribed independently by different coders and then compared. The coding consistency assessed in that way was close to perfect.

2 SUPPLEMENTARY TABLES AND FIGURES

2.1 Sommelier card

EVALUATE THE WINE BY CHOOSING SUITABLE TERMS

Smell of wine

Intensity:
Lacking – scarcely intense – quite intense – intense – very intense

Quality:
Plain – quite fine – excellent

Complexity:
Simple (1-2 fragrance notes sensed) – complex (more than 2 fragrance notes sensed)

Sensed fragrance notes (pick and/or give your own descriptions):
Floral, fruity, herbaceous (ex. grassy, mossy), spicy, mineral, ethereal (chemical), vinous (cellar notes)
Other ____

Comments:____

Taste of wine

Intensity:
Lacking – scarcely intense – quite intense – intense – very intense

Persistence (how long the aftertaste persists in the mouth):
Short – quite persistent – persistent

Quality:
Plain – subtle – excellent

Sugar:
Dry – medium dry – medium sweet – sweet

Acidity:
Flat – quite fresh – fresh – acidulous

Alcohol:
Light – medium – alcoholic

Tannins (roughness, astringent feeling in the mouth):
Lack of tannins (flabby) – scarcely tannic – quite tannic – astringent

Minerality:
Tasteless (bland) – tasty – salty

Structure:
Thin – full – vigorous – heavy

Comments:____

General characteristics of wine:

Smell/Taste harmony:
Disharmonious – harmonious

Evolution:
Young – mature – old (overripe)

Comments:____

Figure S1. Translated sommelier card given to participants.
